# Insectivorous bats respond to vegetation complexity in urban green spaces

**DOI:** 10.1002/ece3.3897

**Published:** 2018-02-19

**Authors:** Marcela Suarez‐Rubio, Christina Ille, Alexander Bruckner

**Affiliations:** ^1^ Institute of Zoology University of Natural Resources and Life Sciences Vienna Vienna Austria

**Keywords:** acoustic monitoring, chiroptera, habitat heterogeneity, mean information gain, vegetation clutter, vegetation complexity

## Abstract

Structural complexity is known to determine habitat quality for insectivorous bats, but how bats respond to habitat complexity in highly modified areas such as urban green spaces has been little explored. Furthermore, it is uncertain whether a recently developed measure of structural complexity is as effective as field‐based surveys when applied to urban environments. We assessed whether image‐derived structural complexity (MIG) was as/more effective than field‐based descriptors in this environment and evaluated the response of insectivorous bats to structural complexity in urban green spaces. Bat activity and species richness were assessed with ultrasonic devices at 180 locations within green spaces in Vienna, Austria. Vegetation complexity was assessed using 17 field‐based descriptors and by calculating the mean information gain (MIG) using digital images. Total bat activity and species richness decreased with increasing structural complexity of canopy cover, suggesting maneuverability and echolocation (sensorial) challenges for bat species using the canopy for flight and foraging. The negative response of functional groups to increased complexity was stronger for open‐space foragers than for edge‐space foragers. *Nyctalus noctula*, a species foraging in open space, showed a negative response to structural complexity, whereas *Pipistrellus pygmaeus*, an edge‐space forager, was positively influenced by the number of trees. Our results show that MIG is a useful, time‐ and cost‐effective tool to measure habitat complexity that complemented field‐based descriptors. Response of insectivorous bats to structural complexity was group‐ and species‐specific, which highlights the need for manifold management strategies (e.g., increasing or reinstating the extent of ground vegetation cover) to fulfill different species’ requirements and to conserve insectivorous bats in urban green spaces.

## 1 INTRODUCTION

Natural ecosystems throughout the world are being subjected to high pressure by human activities. The resulting habitat loss and degradation represent critical threats to biodiversity (e.g., Jones, Jacobs, Kunz, Willig, & Racey, 2009) and potentially affect ecosystem functions and services (Boyles, Cryan, McCracken, & Kunz, 2011). One group that provides essential ecosystem services considered of economic value—such as pollination, seed dispersal, and top‐down control of insects— are bats, the second largest mammalian order (Kunz, Braun de Torrez, Bauer, Lobova, & Fleming, 2011). Bat population declines are mainly ascribed to collisions with wind turbines, the fungal disease white‐nose syndrome, and the reduction, fragmentation, and transformation of natural habitats (Fenton, 1997; Guest, Jones, & John, 2002; Lane, Kingston, & Lee, 2006; O'Shea, Cryan, Hayman, Plowright, & Streicker, 2016). Urbanization, as an example of a severe habitat modification, has strong effects on bats by reducing species richness, altering species composition, and modifying habitat use (e.g., Avila‐Flores & Fenton, 2005; Jung & Kalko, 2011; Kurta & Teramino, 1992; Russo & Ancillotto, 2015).

In natural environments, habitat quality is related to habitat complexity, which in turn is determined by structural complexity of vegetation layers (McElhinny, Gibbons, Brack, & Bauhus, 2005; Tews et al., 2004). In forests, rising habitat complexity results in increased bat species richness (Milne, Fisher, & Pavey, 2006) and higher activity levels (e.g., Frey‐Ehrenbold, Bontadina, Arlettaz, & Obrist, 2013; Jung, Kaiser, Böhm, Nieschulze, & Kalko, 2012) due to its association with available and accessible insect prey (e.g., Grüebler, Morand, & Naef‐Daenzer, 2008; Jung et al., 2012), roosts, and shelter against wind and from predators (Verboom & Spoelstra, 1999). However, some bat species (e.g., open‐space foragers) may avoid forest habitats with dense vegetation when foraging because of morphological and echolocation constraints (Berger & Ehrendorfer, 2011; Brigham, Grindal, Firman, & Morissette, 1997; Müller et al., 2012). In contrast, structurally rich forest habitats are attractive for edge and narrow space foraging bats (e.g., Boughey, Lake, Haysom, & Dolman, 2011; Lesiński, Fuszara, & Kowalski, 2000).

Although urbanization likely represents a serious compromise for many bats (Hale, Fairbrass, Matthews, & Sadler, 2012; Luck, Smallbone, Threlfall, & Law, 2013; Russo & Ancillotto, 2015), urban green spaces (e.g., public parks, reserves, recreation fields and residential gardens) may benefit bats, for example by supplying abundant nocturnal invertebrates (Avila‐Flores & Fenton, 2005) and tree hollows (Basham, Law, & Banks, 2011). However, bats’ response to vegetation complexity of urban green spaces has rarely been characterized (Gehrt & Chelsvig, 2003; Threlfall, Williams, Hahs, & Livesley, 2016), despite their importance for ameliorating the impacts of urbanization and supporting other species (Ives et al., 2016; Park, Mochar, & Fuentes‐Montemayor, 2012). Much of our understanding of the role of vegetation complexity in urban environments has come from studies on urban birds (e.g., Magle, Hunt, Vernon, & Crooks, 2012; Shwartz, Turbe, Julliard, Simon, & Prevot, 2014), but it is unclear whether this understanding is equally relevant to other taxonomic groups (Beninde, Veith, & Hochkirch, 2015). The three‐dimensional arrangement of vegetation may present a range of morphological and sensorial challenges for bats. Thus, understanding specific habitat requirements and bats’ response to vegetation complexity will aid in identifying areas of high conservation priority, in proposing adequate conservation and management strategies, and in forecasting local extinction risks in cities (Threlfall, Law, & Banks, 2012).

Measuring structural complexity from digital images has been recently developed to assess habitat complexity (Proulx & Parrott, 2008, 2009). Image complexity is described by the mean information gain (MIG), which contains spatial and structural information about the represented objects and is a reasonable estimate of habitat complexity in natural environments (Proulx & Parrott, 2008, 2009). Image complexity was successfully used to describe biodiversity patterns of vascular plants in temperate forests (Proulx & Parrott, 2008) and fishes in coral reefs (Mellin et al., 2012). However, it is uncertain whether this method will be useful to assess habitat complexity in urban environments.

The aims of this study were twofold: (1) to assess whether image‐derived structural complexity (MIG) is as/more effective than field‐based descriptors as metric to characterize urban green spaces and (2) to determine whether structural complexity in urban green spaces influences bat activity and species richness. We tested the following hypotheses: (1) measuring structural complexity from digital images complements standard field‐based descriptors. We expected that MIG calculated from images would reflect vegetation parameters recorded in the field and that its inclusion would improve model fit. (2) Bat activity and species richness correlate positively with structural complexity (MIG) because sites with higher vegetation complexity would provide access to resources such as prey and roosts. (3) Functional groups (i.e., edge‐space foragers and open‐space foragers) and representative species (i.e., *Nyctalus noctula, Pipistrellus pygmaeus*) differ in their response to structural complexity. Species foraging at edges or inside vegetation (edge‐space foragers) would show a positive response to structural complexity, whereas species hunting in open space would exhibit a negative response to structural complexity.

## 2 MATERIALS AND METHODS

### 2.1 Study area

**Figure 1:**
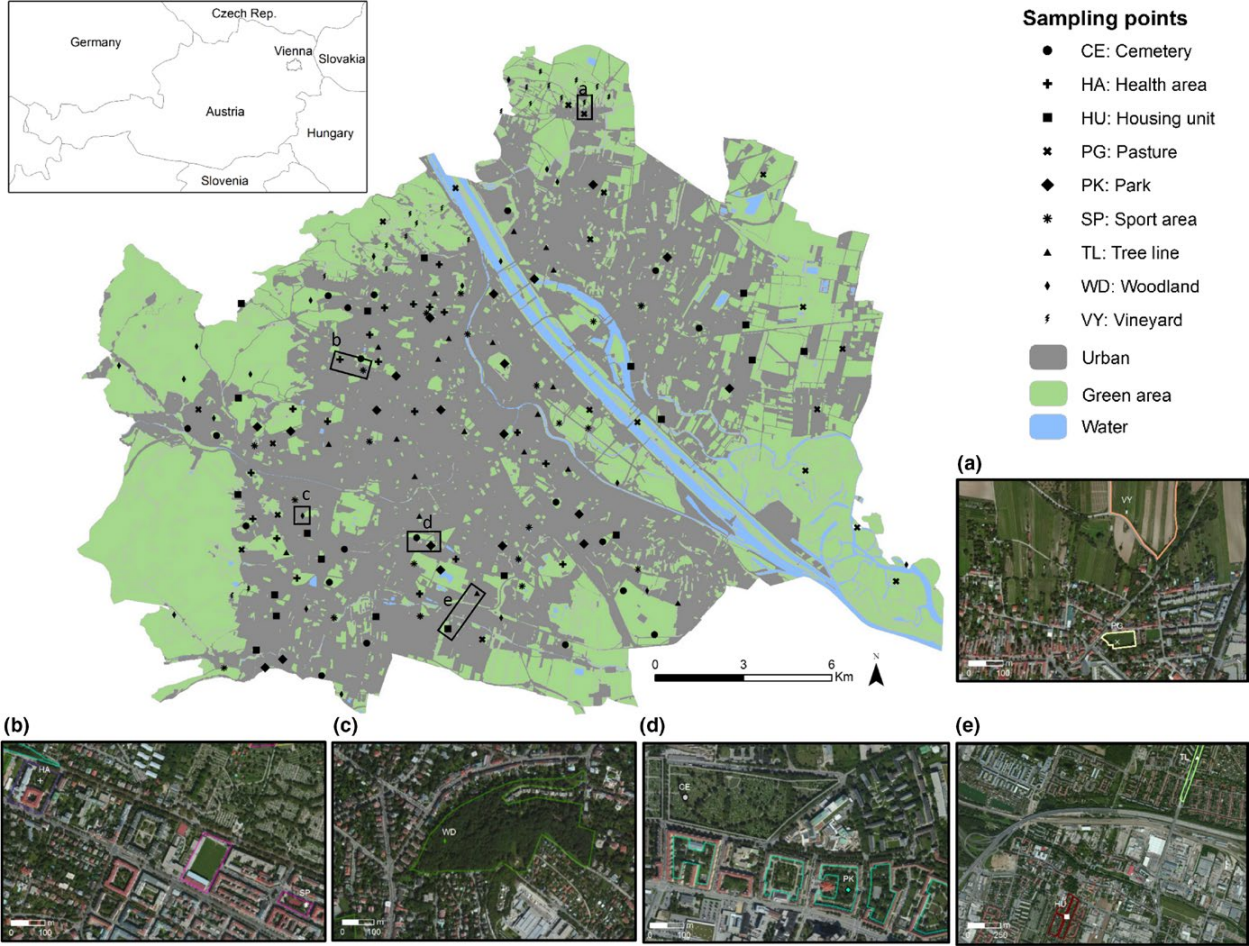
Location of sampling points in Vienna, Austria. Examples of green spaces are depicted in the aerial photos (a) pasture and vineyard, (b) health and sport area, (c) woodland, (d) cemetery and park, (e) housing unit and tree line (Photos courtesy of Google Earth)

The study area was the city of Vienna (48°12′N 16°22′E), Austria (Figure 1), which has a total area of 41,487 ha and elevation ranging between 151 m and 543 m. Being part of the transition zone from the alpine to the continental climate region, Vienna offers a great diversity of vegetation and habitat types (Berger & Ehrendorfer, 2011). In addition, the proportion of green spaces such as parks, residential gardens, and recreation fields is high (~50%) compared to other European metropolises (Hoffert, Fitzka, Stangl, & Lumasegger, 2008). The proportion of green spaces in Vienna ranges from 2%–15% in the inner districts up to 70% in the western part of the city.

### 2.2 Selection of sampling points

A land use map (“Realnutzungskartierung” 2009) by the Vienna municipal department of urban development and planning (MA18) was used as a baseline for selecting the sampling locations. We focused on nine classes referring to green spaces, including cemeteries, health purpose areas, housing units with gardens, parks, sports areas, tree‐lined streets, forests, vineyards, and pastures. Only green spaces larger than 0.25 ha were included. To provide a gradient of size, these areas were divided into quartiles and separated into four different size classes (0–25%, 25–50%, 50–75%, and 75–100%). Five points were randomly chosen for each size class, resulting in 20 data points for each green space type and a total of 180 sampling points (Figure 1). For those green spaces that also contained gray infrastructure (i.e., health purpose areas, housing units with gardens, and sports areas), sampling points were placed in vegetated areas representing the green space (Figure 1). Sampling points were at least 200 m apart (average: 9.6 km).

### 2.3 Structural complexity of digital images

To record the vegetation structure of each sampling point, we took photographs with a commercial digital camera (Nikon Coolpix S220). The images were taken simultaneously with descriptors of vegetation structure measured in the field to insure equal conditions, and always between 09:30 and 15:30 to insure similar illumination. The camera was installed at the center of the sampling point with a tripod placed at a fixed height of 1 m above the ground. Images were taken using automatic mode (focal length 35 mm) and the camera pointing in the four cardinal directions in turn, with the (imaginary) horizon parting the scene in half horizontally. Additionally, one image was taken facing upwards to account for canopy cover.

We converted the RGB color space of all images to hue, saturation, value (HSV) to separate the pure color component (hue) from chroma (saturation) and intensity (value) as suggested by Proulx and Parrott (2008). Conversion is necessary given the considerable overlap of transmittances among the three spectral bands (RGB), which is not present in the HSV space. Additionally, HSV reproduces more effectively how the human brain represents color, without information loss of within‐ and among‐image variation in the RGB color space (Mellin et al., 2012). We used the V‐layer of the images for analysis, which Proulx and Parrott (2008, 2009) have identified as a robust value for quantifying structural complexity in natural scenes.

We used mean information gain (MIG) to quantify structural complexity because it is a well‐established measure of spatial complexity patterns (e.g., Gell‐Mann & Lloyd, 1996; Wackerbauer, Witt, Atmanspacher, Kurths, & Scheingraber, 1994). Mean information gain (MIG) was calculated based on Shannon′s equation for entropy using the amount of spatial heterogeneity in an image (i.e., joint entropy) and the fraction of aspatial heterogeneity (i.e., marginal entropy) (Proulx & Parrott, 2008, 2009). Mean information gain (MIG) for the intensity band (V) in the HSV image was computed as:

(1) $$\text{MIG}=\frac{\left[-\sum_{j=1}^{M^{k}}p\left(\chi_{j}\right)\text{log}p\left(\chi_{j}\right)\right]-\left[-\sum_{i=1}^{M}p\left(\gamma_{i}\right)\text{log}p\left(\gamma_{i}\right)\right]}{\text{log}(M^{k}/M)}$$

where *p (*χ_*i*_ *)* is the probability of finding a specific spatial configuration χ_*i*_ made of *k* neighboring pixels in the image (*k *=* *4 representing a 2 × 2‐pixel neighborhood). *p* (γ_*i*_) denotes the probability of observing a pixel′s intensity value γ_*i*_ independently of its location in the image. *M* is the number of frequency bins of pixel values, and *M* ^*k*^ represents the maximum number of possible pixel configurations in a four‐pixel neighborhood. To retain as much image information as possible, we chose the highest possible value of *M*.

Mean information gain (MIG) ranges from zero, for completely uniform spatial patterns across pixels (order), which would represent a single color, to one for completely random patterns (disorder) (Proulx & Parrott, 2008, 2009). Thus, images of undifferentiated, uniform habitats have low MIG, while images of random or highly differentiated habitats have high MIG. Intermediate values of MIG are associated with more spatially heterogeneous data, hence, higher habitat complexity (Parrott, 2010). Mean information gain (MIG) was processed for each of the five images per sampling point. We calculated the mean to combine the images per sampling location into one index as suggested by Proulx and Parrott (2008) and Mellin et al. (2012). MIG of all five images (MIG all views) was used as an individual parameter, as was MIG of side views only (MIG sides) and MIG of the top view only (MIG top). All digital images were processed with R v. 3.2.3 (R Core Team, 2015) using the “imagemetrics” package v. 1.0 (Massicotte, 2014).

### 2.4 Field‐based descriptors of vegetation structure

From June to September 2014, we recorded vegetation variables in a 20‐m radius plot centered at each sampling point. Vegetation variables recorded were as follows: vegetation profile (vertical layering of vegetation), ground cover (vegetation cover on the ground), canopy cover, vegetation height, tree density, and number and diameter at breast height of large trees (DBH > 0.30 m). To determine vegetation profile, ground cover, canopy cover, and vegetation height, we used the cover‐board method (Nudds, 1977) as a baseline. We positioned a 2‐m vertical pole, marked every 0.5 m, at the center point and at four points at 5‐m interval along the cardinal directions and recorded the presence or absence of foliage touching the following height classes: 0–0.5, 0.5–1, 1–1.5, 1.5–2, 2–3, 3–5, 5–7, 7–10, 10–15, and >15 m above ground. For height intervals above 2 m, we estimated the height and recorded presence/absence of foliage. The average of vegetation touches per height class was used as a proxy of vegetation clutter for each height. Additionally, we combined all vegetation classes to calculate vertical heterogeneity using the Shannon diversity index in which the number of vegetation touches of the 17 measurement positions using the measuring pole at each height interval is equivalent to individuals for this height class (Sekercioglu, 2002). The index was calculated with R using the “vegan” package v. 2.3‐0 (Oksanen et al., 2015). In each of the measurement positions, we estimated the proportion of vegetation covering the ground, the percentage of canopy cover, and the vegetation height. We calculated tree density of trees with DBH > 0.16 m for the whole plot, counted large trees (DBH > 0.3 m), and measured their DBH. These variables were selected to characterize vegetation‐structural complexity and clutter at different vegetation heights and for comparison with the image‐derived structural complexity measures.

### 2.5 Acoustic bat sampling and call analysis

Bat surveys were conducted from the end of April to the end of September 2014. We repeated recordings three times at each sampling point, resulting in 540 recording nights. Bat vocalizations were digitally recorded with ultrasound devices (Batcorder 1.0 and 2.0, ecoObs, Nuremberg, Germany) which were placed on an attachment rod of approximately 2‐m height and at least 2 m away from any structure in each direction. The manufacturer's default settings (quality 20, threshold—27 db, posttrigger 600 ms, critical frequency 16 kHz) were used, and a fully automatic recording was started 1 hr before sunset and ended 1 hr after sunrise. Surveys were only conducted during suitable weather conditions (i.e., >10°C, no rain or strong winds). Although acoustic sampling may misrepresent bats that use echolocation calls of lower intensity (e.g., *Plecotus* spec.) or be influenced by habitat features (Patriquin, Hogherg, Chruszcz, & Barclay, 2003; Schnitzler & Kalko, 2001), we are confident that, by sampling the entire night and using a standardized and replicated sampling scheme (Froidevaux, Zellweger, Bollmann, & Obrist, 2014; Skalak, Sherwin, & Brigham, 2012), we registered the majority of species and most of the activity in the green spaces.

Species identification was performed using automated systems as they provide a standardized identification and is less time‐consuming than manual identification especially when many recordings are available (Jennings, Parsons, & Pocock, 2008). We processed the recordings with the software bcAdmin v. 2.12, batIdent v. 1.03, and bcAnalyze v. 1.16 (ecoObs. Nuremberg, Germany). bcAdmin discriminates bat calls from the sound file and measures their acoustic properties (e.g., call length). batIdent uses the calls’ acoustic properties and a classification tree algorithm to identify species. bcAnalyze depicts sonograms and allows extended playbacks of bat calls to manually inspect dubious call sequences (i.e., false positive IDs). Given the concerns raise about the reliability of various automated identification programs (Russo & Voigt, 2016; Rydell, Nyman, Eklöf, Jones, & Russo, 2017), manual validation of the output of the automated identification was performed by an expert (AB), as the degree of experience can bias the validation process (Fritsch & Bruckner, 2014). Dubious identifications were critically evaluated using call descriptors; geographic, altitudinal, and habitat preferences of the suggested species; and the guidelines by Hammer and Zahn (2009). The sounds of some species could not be reliably discerned; thus, we combined the following species to so‐called “acoustic groups”: *Pipistrellus kuhlii* and *Pipistrellus nathusii*;* Myotis mystacinus* and *Myotis brandtii*;* Plecotus auritus* and *Plecotus austriacus*. For simplicity, actual species and acoustic groups are referred to as “species” hereafter. Bat activity was defined as the median length of the call sequences for the three surveys and was highly correlated with the number of calls (*r* = .9). Species richness was defined as the total number of species recorded during all three surveys.

### 2.6 Statistical analysis

We used Pearson′s correlation coefficient (|*r*| *>* .5) to assess the relationship between MIG and field‐based descriptors (vegetation clutter at different heights, ground cover, canopy cover, vegetation height, vertical heterogeneity, tree density, and number and DBH of large trees), and between MIG and bat activity and species richness.

Analyses were performed for total bat activity, total species richness, and the following functional groups according to Schnitzler and Kalko (2001) and Denzinger and Schnitzler (2013): edge‐space foragers (*P. pipistrellus, P. pygmaeus, P. kuhlii/P. nathusii, Barbastella barbastellus, Myotis alcathoe, M. brandtii/M. mystacinus*) and open‐space foragers (*N. noctula, Eptesicus nilssonii, Hypsugo savii*). To examine potential differences between individual species, activity of *N. noctula* (the largest western and central European bat and an open‐space forager) and *P. pygmaeus* (a small bat and an edge‐space forager) was analyzed as representative species.

Exploratory analyses revealed that average night temperature and length of the night had no correlation with any of the response variables (Spearman's Rho < 0.2 in all cases); hence, these variables were not considered further. Because bat activity (but not species richness) varied between different green space types, we included green space type when modeling bat activity. Bat activity was log‐transformed, and continuous predictor variables were standardized to have a mean of zero and standard deviation of one. Variance inflation factor (VIF) was used to identify multicollinearity (VIF > 2) using the “usdm” package v. 1.1‐15 (Naimi, 2015). As predictor variables, in addition to size of green space, we included only MIG parameters, only field‐based descriptors, and both combined. When MIG was included, we either used MIG all views or MIG sides and MIG top separately, as they potentially contain information with different relevance for bats.

We conducted generalized linear mixed models with Gaussian error distribution to analyze which variables influenced bat activity using the function lmer from the “lme4” package v. 1.1‐12 (Bates, Maechler, Bolker, & Walker, 2015). Green space type was included as a random factor to account for multiple samples within each green space type. We used generalized linear Poisson models to determine the best predictors of bat species richness. Model selection was performed using a stepwise procedure and Akaike's information criterion (AIC; package “MASS” v. 7.3‐45) (Venables & Ripley, 2002). A model with the smallest AIC value was considered the best‐fitting model. Where Poisson GLM revealed overdispersion, correction of standard errors using a quasi‐Poisson GLM model was conducted (function: glm, family: quassipoisson) (Zuur, Ieno, Walker, Saveliev, & Smith, 2009). We validated the model by assessing homoscedasticity and independence. All statistical analyses were performed in R v. 3.2.3 (R Core Team, 2015).

## 3 RESULTS

### 3.1 Structural complexity derived from digital images

Mean information gain (MIG) was correlated with several field‐based descriptors (Figure S1). Mean information gain (MIG) all views were correlated with canopy cover (*r* = .68), tree density (*r* = .64), vertical heterogeneity (*r* = .58), vegetation height (*r* = .64), and number of trees (*r* = .54). MIG sides were correlated with vertical heterogeneity (*r* = .54), whereas MIG top was correlated with canopy cover (*r* = .62) and vegetation height (*r* = .54).

There was no correlation between MIG all views and bat activity (*r* = −.07) or species richness (*r* = −.18), nor was there a correlation between MIG sides and bat activity (*r* = .12) or species richness (*r* = .03). A slight negative correlation was found between MIG top and both bat activity (*r* = −.33) and species richness (*r* = −.39, Figure S2).

**Figure 2:**
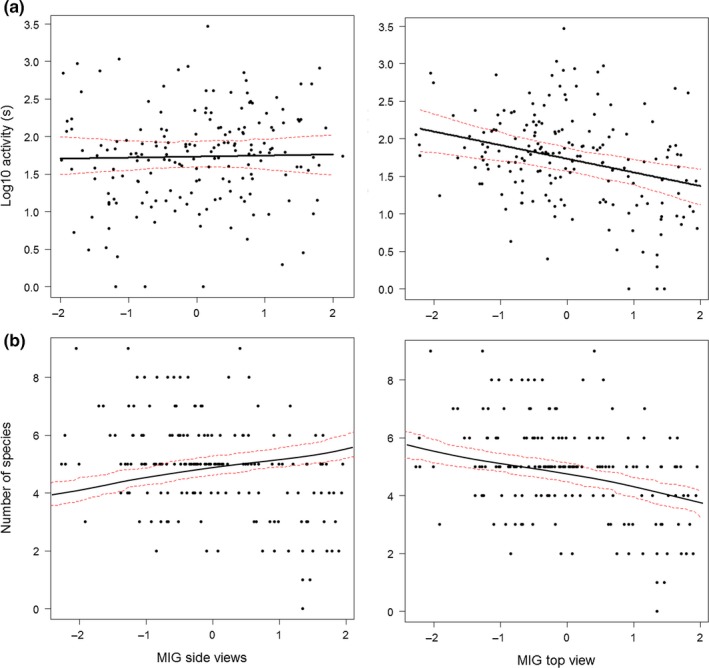
Regression‐fitted lines with 95% confidence intervals for (a) total bat activity, (b) species richness as a function of structural complexity parameters derived from digital images showing horizontal (MIG side views on left panels) or vertical view (MIG top view on right side panels). Explanatory variables (except the one varying on the *x*‐axes) were set to their mean values

When only MIG parameters were considered in our generalized linear mixed models as potential predictors of bat activity and species richness (Table 1, Figure 2), MIG top had a significant negative effect on total bat activity, activity of edge‐space foragers, open‐space foragers and *N. noctula*, whereas MIG sides had a significant positive effect on activity of *P. pygmaeus* (Figure S3). Similarly, MIG top had a significant negative effect on total species richness and species richness of edge‐ and open‐space foragers (Table 1).

**Table 1:** Summary of results of generalized linear mixed models including green space type as a random factor and generalized linear Poisson models for bat activity, species richness, edge‐ and open‐space foragers, and representative species (*Nyctalus noctula* and *Pipistrellus pygmaeus*) as a function of only MIG parameters . Significant *p*‐values are shown in bold

	Estimate	*SE*	*t*‐value	*p*	Pseudo‐*R* ^2^
Total bat activity					
MIG sides	0.014	0.046	0.306	.760	.25
MIG top	−0.182	0.045	−4.008	**<.001**	
Activity edge‐space foragers					
MIG sides	0.049	0.055	0.897	.371	.18
MIG top	−0.178	0.054	−3.269	**<.001**	
Activity open‐space foragers					
MIG sides	−0.039	0.057	−0.692	.490	.27
MIG top	−0.295	0.056	−5.240	**<.001**	
Activity *N. noctula*					
MIG sides	−0.071	0.053	−1.347	.180	.23
MIG top	−0.249	0.053	−4.733	**<.001**	
Activity *P. pygmaeus*					
MIG sides	0.251	0.126	1.991	**.048**	.09
MIG top	−0.054	0.125	−0.432	.667	
Total species richness					
MIG sides	0.015	0.023	0.625	.533	.14
MIG top	−0.130	0.023	−5.551	**<.001**	
Species richness edge‐space foragers					
MIG sides	0.029	0.026	1.109	.269	.03
MIG top	−0.063	0.026	−2.374	**.019**	
Species richness open‐space foragers					
MIG sides	−0.014	0.029	−0.473	.637	.17
MIG top	−0.209	0.030	−7.023	**<.001**	

### 3.2 Influence of MIG and field‐based descriptors on bat activity

**Figure 3:**
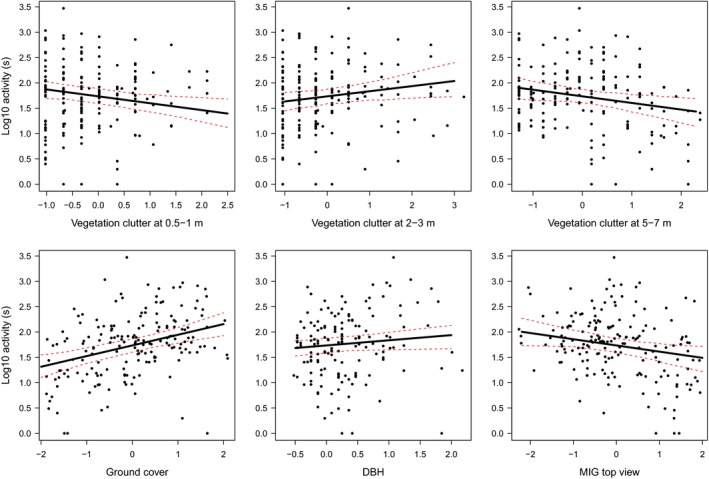
Regression‐fitted lines with 95% confidence intervals for total bat activity when both field‐based descriptors and structural complexity parameters were considered. All explanatory variables (except the one varying on the *x*‐axes) were set to their mean values. Only significant variables are shown

Canopy cover, vegetation height, tree density, and vegetation clutter at 10–15 m were highly collinear with number of trees; as were vegetation clutter at 0–0.5, 1–1.5, and 1.5–2 m with vegetation clutter at 0.5–1 m; vegetation clutter at 2–3 m with vegetation clutter at 3–5 m; and vegetation clutter at 3–5 and 7–10 m with vegetation clutter at 5–7 m. Thus, variables that were included in generalized linear mixed models were vegetation clutter at 0.5–1, 2–3, 5–7, >15 m, ground cover, vertical heterogeneity, number of trees, and DBH. When evaluating the influence of only the field descriptors, total bat activity decreased significantly with vegetation clutter at 0.5–1 m and 5–7 m, while it increased with ground cover, vegetation clutter at 2–3 m, and DBH (Table 2). When MIG was included, MIG top was also a significant negative predictor, and the explained variation increased slightly (Table 2, Figure 3).

**Table 2:** Summary of results of generalized linear mixed models including green space type as a random factor and generalized linear Poisson models for bat activity and species richness including edge‐ and open‐space foragers, and representative species (*Nyctalus noctula* and *Pipistrellus pygmaeus*) as a function of only field‐based descriptors and including MIG

	Only field‐based descriptors					Including MIG parameters				
	Estimate	*SE*	*t*‐value	*p*	Pseudo‐*R* ^2^	Estimate	*SE*	*t*‐value	*p*	Pseudo‐*R* ^2^
Total bat activity										
Vegetation clutter at 0.5–1 m	−0.134	0.047	−2.870	.005	.30	−0.136	0.046	−2.946	.004	.33
Vegetation clutter at 2–3 m	0.109	0.049	2.240	.026		0.101	0.048	2.105	.037	
Vegetation clutter at 5–7 m	−0.186	0.050	−3.752	<.001		−0.130	0.054	−2.433	.016	
Ground cover (%)	0.234	0.049	4.829	<.001		0.210	0.049	4.315	<.001	
DBH	0.097	0.046	2.108	.037		0.102	0.045	2.242	.026	
MIG top						−0.122	0.048	−2.531	.012	
Activity edge‐space foragers										
Ground cover (%)	0.159	0.056	2.855	.005	.14	0.144	0.056	2.552	.012	.17
MIG top						−0.126	0.053	−2.366	.019	
Activity open‐space foragers										
Vegetation clutter at 5–7 m	−0.239	0.057	−4.218	<.001	.41	−0.197	0.061	−3.215	.001	.41
Ground cover (%)	0.248	0.056	4.442	<.001		0.232	0.056	4.129	<.001	
Size green space	−0.138	0.048	−2.826	.005		−0.129	0.048	−2.651	.008	
MIG top						−0.116	0.058	−2.020	.045	
Activity *N. noctula*										
Vegetation clutter at 0.5 – 1 m	−0.076	0.037	−2.053	.042	.39	−0.079	0.036	−2.208	.029	.40
Vegetation clutter at 5–7 m	−0.259	0.048	−5.382	<.001		−0.209	0.054	−3.893	<.001	
Ground cover (%)	0.301	0.053	5.603	<.001		0.279	0.054	5.164	<.001	
MIG top						−0.109	0.054	−2.009	.046	
Activity *P. pygmaeus*										
No. trees	0.403	0.113	3.553	<.001	.16	0.403	0.113	3.553	<.001	.16
Ground cover (%)	0.597	0.113	5.256	<.001		0.597	0.113	5.256	<.001	
Total species richness										
Vegetation clutter at 5–7 m	−0.094	0.023	−4.104	<.001	.21	−0.062	0.026	−2.376	.019	.24
Ground cover (%)	0.115	0.022	5.130	<.001		0.101	0.023	4.394	<.001	
MIG top						−0.068	0.027	−2.519	.013	
Species richness edge‐space foragers										
Ground cover (%)	0.080	0.026	3.080	.002	.05	0.080	0.026	3.080	.002	.05
Species richness open‐space foragers										
Vegetation clutter at 5–7 m	−0.151	0.030	−4.993	<.001	.25	−0.151	0.030	−4.993	<.001	.25
Vegetation clutter above 15 m	−0.131	0.040	−3.284	.001		−0.131	0.040	−3.284	.001	
Ground cover (%)	0.131	0.029	4.551	<.001		0.131	0.029	4.551	<.001	

**Figure 4:**
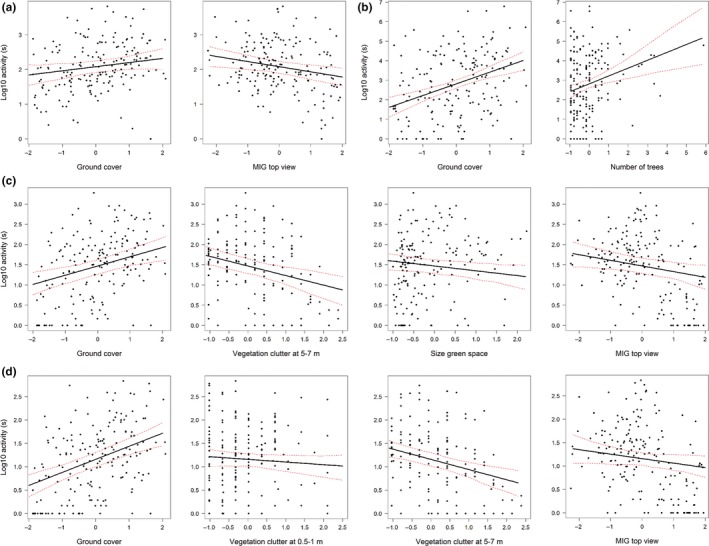
Regression‐fitted lines with 95% confidence intervals for activity of (a) edge‐space foragers, (b) *Pipistrellus pygmaeus* (c) open‐space foragers, and (d) *Nyctalus noctula* when both field‐based descriptors and structural complexity parameters were considered. All explanatory variables (except the one varying on the *x*‐axes) were set to their mean values. Only significant variables are shown

Activity of edge‐space foragers was only positively influenced by ground cover (Table 2). When MIG parameters were included, MIG top had an additional significant negative influence (Figure 4) and the explained variation increased slightly (*R*² only field parameters = .14, including MIG parameters = 0.17; Table 2). Besides the positive influence of ground cover, activity of open‐space foragers decreased with vegetation clutter at 5–7 m and size of green space when evaluating only field variables (Table 2). When MIG was included, MIG top also had a negative effect (Figure 4).

Activity of *N. noctula* was negatively affected by vegetation clutter at 0.5–1 and 5–7 m and positively influenced by ground cover (Table 2). Inclusion of MIG top had a significant negative impact (Figure 4), and the explained variation was similar. Besides ground cover, activity of *P. pygmaeus* also increased with the number of trees. Mean information gain (MIG) parameters had no effect on activity of *P. pygmaeus* (Table 2, Figure 4).

### 3.3 Influence of MIG and field‐based descriptors on species richness

We recorded 11 bat species and three acoustic groups of which the three most common were *P. pygmaeus, P. kuhlii*/*P. nathusii* occurring at 93% of all sampling points, followed by *N. noctula (*88%), *H. savii* (73%), and *P. pipistrellus* (61%). The acoustic group *M. mystacinus/M. brandtii* was relatively common, occurring at one‐third of the sites. Six other species were detected at <10% of the sites (Table A1).

Total species richness and species richness of open‐space foragers showed similar responses to vegetation complexity (Table 2). Ground cover had a significant positive influence on species richness, whereas vegetation clutter at 5–7 m had a negative influence. In addition, vegetation clutter at above 15 m had a negative influence on species richness of open‐space foragers. When MIG parameters were included, the explained variation increased slightly for total species richness (Table 2) and MIG top had an additional negative effect. In contrast, incorporating MIG parameters did not have any effect on species richness of edge‐space foragers which were only positively affected by ground cover (Table 2).

## 4 DISCUSSION

We evaluated the suitability of a structural complexity measure derived from digital images (MIG) as a useful ecological tool to describe vegetation complexity in an urban environment and assessed the response of insectivorous bats to structural complexity of urban green spaces.

### 4.1 Image‐derived structural complexity vs. field‐based descriptors

Image‐derived structural complexity (MIG) complemented field‐based descriptors and captured vegetation patterns in urban green spaces, although models conducted with only field‐based data generally showed higher explanatory power compared to models that only used MIG. There are different possible explanations for these results. MIG was able to depict vegetation structure (e.g., canopy cover); indeed, MIG top view replaced canopy cover and vegetation height due to high collinearity (Figure S1). Although MIG aggregated individual characteristics of structural complexity into a single measure, the influence of single attributes (i.e., canopy complexity and structural complexity) was distinguishable (Figure 2). However, it is possible that anthropogenic structures in urban environments influence the performance of MIG. Digital images took every structure of the scenery into account, whereas data collected with the field‐based method only considered vegetation. If and to what extent anthropogenic structures change image complexity remains to be further investigated, but any such effect may depend on their size, form, frequency, and distance from the camera.

Nonetheless, the strength of structural complexity derived from digital images was apparent. MIG parameters analyzed separately had considerable explanatory power. It is remarkable that models performed solely with MIG parameters accounted for up to one‐fourth of the variation explained, given the relatively small effort involved in their sampling. While photographs were taken in a very brief time period (5 min per site), recording field‐based descriptors lasted 1 hr on average and up to 2 hr in places with high vegetation complexity. In addition, processing the images and calculation of MIG took 5 min per site (1 min per image), whereas processing field data required an average of 40 min per site. This adds up to a saving of +270 hr and makes MIG an efficient way to sample a large number of sites.

Besides being labor intensive, field‐based descriptors may be limited in their ability to describe important structural complexity attributes, given the variety of possible measures and the need to consider a suite of attributes simultaneously to reflect the inherent characteristics of a site (McElhinny et al., 2005). Thus, MIG is an objective descriptor of structural complexity that does not require a subjective selection of specific structural attributes. However, it is important to note that MIG uses the spatial distribution of the colors of pixels in a two‐dimensional space to represent complex patterns that emerge from the presence and the spatial associations of three‐dimensional objects. Although MIG correlates with three‐dimensional metrics (e.g., fractal dimension) (Proulx & Parrott, 2009), further research is required to compare MIG to other approaches that depict space in three‐dimensions (e.g., Light Detection and Ranging [LiDAR]).

In summary, our results suggest that MIG as a complexity measure is a useful and time‐efficient tool for characterizing vegetation patterns in urban green spaces. However, MIG may be less informative when knowledge about amount of vegetation—not only complexity—is required. Field‐based descriptors, on the contrary, may be more suitable when the aim is to link species patterns to certain vegetation structures as they allow identifying ecological mechanisms. To improve MIG, we recommend taking photographs also from the outer perimeter of the plots inwards, for example, or by pointing the camera upwards at different angles, similar to the way field‐based descriptors were recorded.

### 4.2 Response of insectivorous bats to structural complexity

Our results indicate that, in most cases, canopy complexity (MIG top view) of urban green spaces had a significant negative effect on bats. This effect remained even when field‐based descriptors were included. The negative effect of canopy complexity in green spaces indicates that areas with open canopy may facilitate foraging activity for some bat species. This is supported by previous findings where bat activity decreased with canopy closure and increased with relative area of open canopy (Ford, Menzel, Menzel, Edwards, & Kilgo, 2006; Kusch, Weber, Idelberger, & Koob, 2004). Foliage‐free spaces between tree crowns were also more suitable for hunting bats, as these combined high insect abundance and the obstacle‐free spaces needed for foraging, while still providing some protection from aerial predators (Marques, Ramos Pereira, & Palmeirim, 2016). Canopy complexity, therefore, seems to be disadvantageous to the majority of recorded species likely due to maneuverability and echolocation characteristics (Kusch et al., 2004; Müller et al., 2012). One could also argue that the negative effect of canopy complexity may be an artifact given that bat calls might be easier to detect in areas with low vegetation clutter. However, we insured that the vicinity of bat detectors was free of any structures to minimize this known bias. In addition, we believe that we further coped with potential biases by analyzing different functional groups and representative species.

Species richness was not affected by structural complexity (MIG side views), which was surprising because complex habitats comprise more niches and would therefore be expected to increase the number of species (e.g., Huston, 1979). To date, only a few studies have demonstrated a positive relationship between image‐derived habitat complexity and species richness in natural environments (Mellin et al., 2012; Proulx & Parrott, 2008). In this study, only canopy complexity had a negative effect on total species richness, which highlights that canopy vegetation patterns are a key factor affecting the number of species in green spaces. However, given the low explained variability, other factors such as proportion of native vegetation (Threlfall et al., 2016) or anthropogenic disturbance (Stone, Harris, & Jones, 2015) may potentially have a greater impact than habitat or canopy complexity alone.

Previous studies have suggested that vegetation clutter at 10‐20 cm seems to reduce access to prey, affecting both capture success and time to capture (Rainho, Augusto, & Palmeirim, 2010). However, we found that proportion of vegetation cover on the ground had a positive effect on bat activity and species richness, even when MIG variables were included. Given the high degree of imperviousness in cities, vegetation on the ground in green spaces may provide higher insect density (Di Giulio, Edwards, & Meister, 2001; Grüebler et al., 2008). Nonetheless, vegetation clutter at 0.5‐1 m indeed decreased total bat activity, suggesting the adverse effect of clutter in the understory (e.g., Brigham et al., 1997; Law & Chidel, 2002; Lintott et al., 2015).

As expected, vegetation clutter at different heights affected activity of edge‐ and open‐space foragers in different ways. Edge‐space foragers are adapted to hunting in gaps but in the vicinity of background clutter (Adams, Law, & French, 2009; Denzinger & Schnitzler, 2013), whereas open‐space foragers and *N. noctula*, a typical open‐space bat, were negatively affected by vegetation clutter. Open‐space foragers show higher avoidance of closed canopy areas than edge‐space foragers because of their different physiological tolerances to structural clutter (Crome & Richards, 1988) and different hunting strategies (Denzinger & Schnitzler, 2013). Nonetheless, open‐space foragers may use closed forest, but above the tree canopy (Müller et al., 2013). *Nyctalus noctula*, in particular, flies at heights of up to 100 m, but hunts within a range of 5–20 m (Richarz & Limbrunner, 1992). Therefore, clutter in the upper layers and tree canopy may be unfavorable.

Interestingly, open‐space foragers were highly active in small green spaces. Perhaps this can be explained by their flexibility in echolocation behavior (Rydell, 1990) or the synanthropization (Uhrin et al., 2016) of some of the species in this group. When flying in the open space above roofs or vegetation, these species predominantly use their long, almost‐constant‐frequency calls. If they use the more limited space in small green spaces (i.e., more clutter due to neighboring buildings), then open‐space foragers may “dive down” into them and thus modify the structure of their calls by switching to shorter, more frequency‐modulated calls to achieve a higher resolution of obstacles. Alternatively, some species have adapted to urban environments (Uhrin et al., 2016) and use buildings as daytime roosts (Kubista & Bruckner, 2015); thus, higher activity in small green spaces could be associated with accessing roosting sites or searching for new locations.

In contrast to the other groups, activity of *P. pygmaeus* increased significantly with the number of trees (DBH > 0.3 m). This result supports previous findings that *P. pygmaeus* uses urban woodlands or seminatural habitats (Glendell & Vaughan, 2002; Lintott et al., 2015; Park et al., 2012). The species’ high maneuverability and hunting strategy (i.e., within or near vegetation) explain the tolerance of *P. pygmaeus* to vegetation clutter and thus the lack of significance of any structural complexity variables. Besides providing potential prey, trees also offer shelter from wind and predation (Verboom & Spoelstra, 1999) and indeed are valuable habitat components for foraging insectivorous bats (Lumsden & Bennett, 2005).

## 5 CONCLUSIONS

Our results suggest that image‐derived structural complexity (MIG) is a time‐ and cost‐effective tool for capturing complexity of vegetation and complements field‐based descriptors. No method is likely to meet all needs. When time and money are a serious constraint and research questions do not aim to identify underlying ecological mechanisms, MIG is a suitable sampling tool. Otherwise, other measures such as field‐based descriptors should be considered.

We demonstrated that insectivorous bats respond to structural complexity in urban green spaces. Additionally, a proportion of vegetation covering the ground and number of large trees favor total bat activity. Although canopy complexity had a negative effect in most cases, bats’ response to structural complexity was group‐ and species‐specific. Open‐space foragers as well as *N. noctula* showed a higher avoidance of vegetation clutter at the upper layers and tree canopy than edge‐space foragers. In contrast, *P. pygmaeus*, an edge‐space forager, was positively influenced by the number of trees.

The group‐ and species‐specific response to vegetation‐structural complexity highlights the need for manifold management strategies in urban green spaces: increasing or reinstating the extent of ground vegetation cover, including seedlings and grass; managing complex vegetation in multiple strata, such as promoting shrubs and bushes while simplifying higher strata; or balancing out areas of high complexity with areas encompassing shorter and simplified vegetation and open spaces. Lastly, retaining large and mature trees would support sensitive species. These strategies would insure conservation of the full spectrum of insectivorous bat species occurring in urban green spaces.

## CONFLICT OF INTEREST

The authors declare that they have no competing interests.

## AUTHORS’ CONTRIBUTIONS

MSR and AB conceived the ideas; MSR designed the study; CI collected the data; MSR, CI, and AB analyzed the data; MSR and CI led the writing of the manuscript. All authors contributed critically to the drafts and approved the final manuscript.

## DATA ACCESSIBILITY

Data available on Dryad Digital Repository: https://doi.org/10.5061/dryad.5g52t

## APPENDIX 1

### 1.1

**Table A1:** Occurrence of verified bat species recorded in 180 sampling points in Vienna, Austria

Species	Common name	Acronym	Occurrence (%)	FFH‐directive	Red List
*Pipistrellus pygmaeus*	Soprano pipistrelle	Ppyg	93.3	IV	DD
*Pipistrellus kuhlii*	Kuhl's pipistrelle	Pmid	93.3	IV	VU
*Pipistrellus nathusii*	Nathusius’ pipistrelle	Pmid	93.3	IV	NE
*Nyctalus noctula*	Common noctule	Nnoc	88.3	IV	NE
*Hypsugo savii*	Savi's pipistrelle	Hsav	73.3	IV	EN
*Pipistrellus pipistrellus*	Common pipistrelle	Ppip	61.1	IV	NT
*Myotis mystacinus*	Whiskered bat	Mbart	31.7	IV	NT
*Myotis brandtii*	Brandt's bat	Mbart	31.7	IV	VU
*Barbastella barbastellus*	Barbastelle	Bbar	18.9	II, IV	VU
*Plecotus auritus*	Brown/Common long‐eared bat	Plecotus	16.1	IV	VU
*Plecotus austriacus*	Gray long‐eared bat	Plecotus	16.1	IV	LC
*Myotis daubentonii*	Daubenton's bat	Mdau	3.9	IV	LC
*Myotis emarginatus*	Geoffroy's bat	Mema	3.3	II, IV	VU
*Myotis nattereri*	Natterer's bat	Mnat	3.3	IV	VU
*Eptesicus nilssonii*	Northern bat	Enil	2.2	IV	LC
*Myotis myotis*	Greater mouse‐eared bat	Mmyo	1.7	II, IV	LC
*Myotis alcathoe*	Alcathoe Whiskered bat	Malc	0.6	IV	–

Scientific and common names, acronyms, and conservation status of the FFH‐directive and of the Red list of Austrian′s endangered mammals (Spitzenberger 2005) are included: EN (endangered), VU (vulnerable), NT (near threatened), LC (least concern), DD (data deficient), NE (not evaluated),— (not listed).
