# Supplementary figures and images for: Insectivorous bats respond to vegetation complexity in urban green spaces
Source: Ecol Evol. 2018 Feb 19;8(6):3240–53. doi: 10.1002/ece3.3897 (PMC5869212; doi:10.1002/ece3.3897)

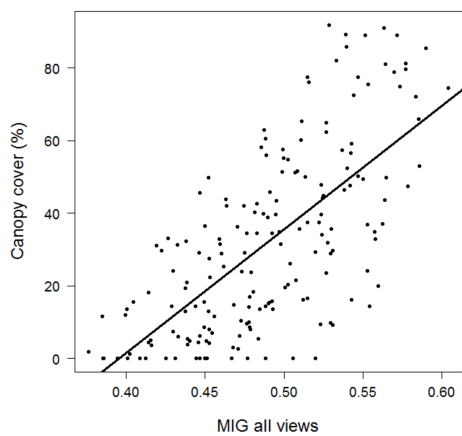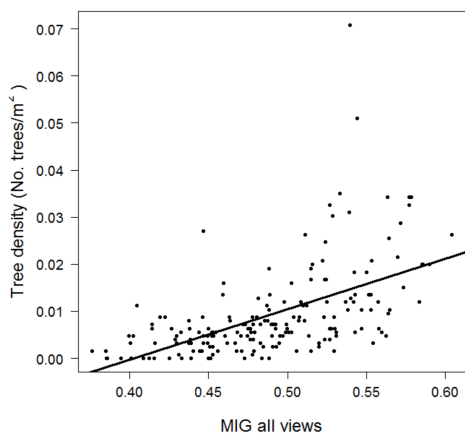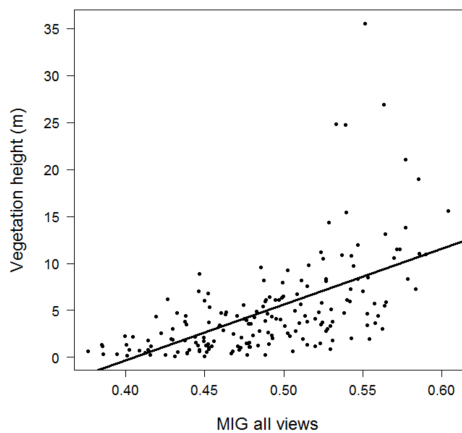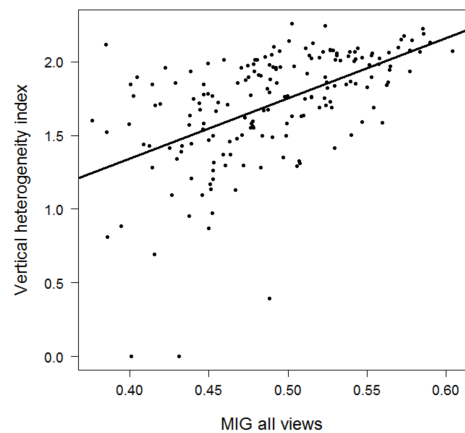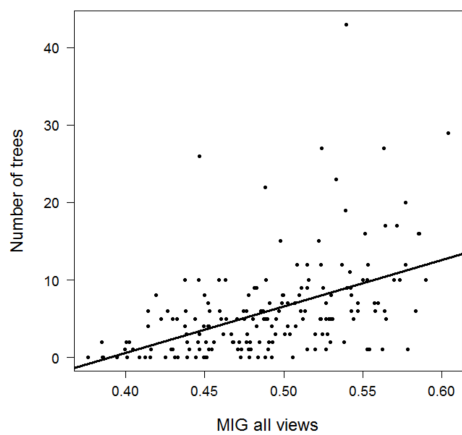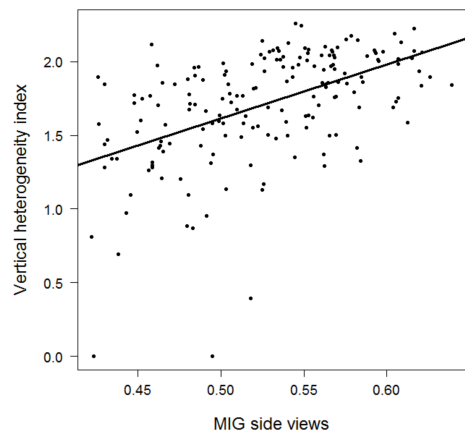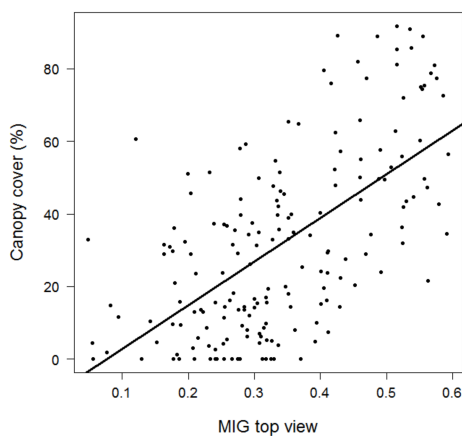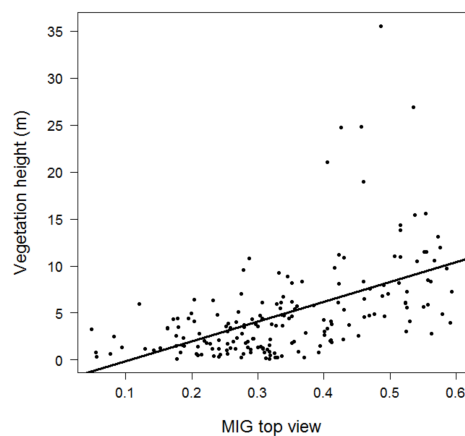

Supplement: Supplementary file 1 [file ECE3-8-3240-s001.pdf]

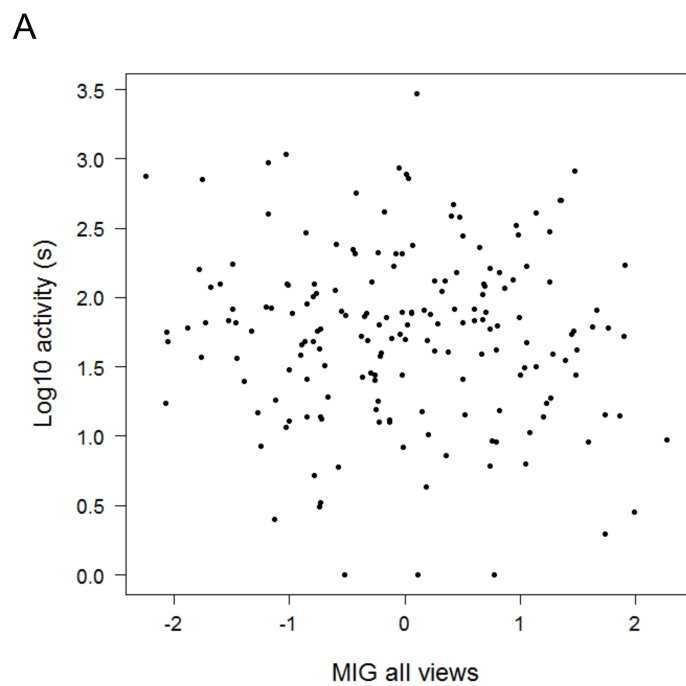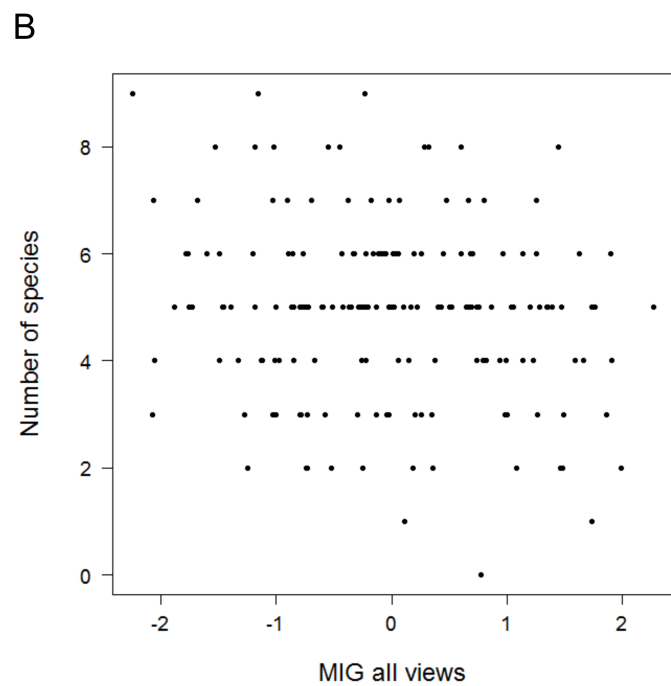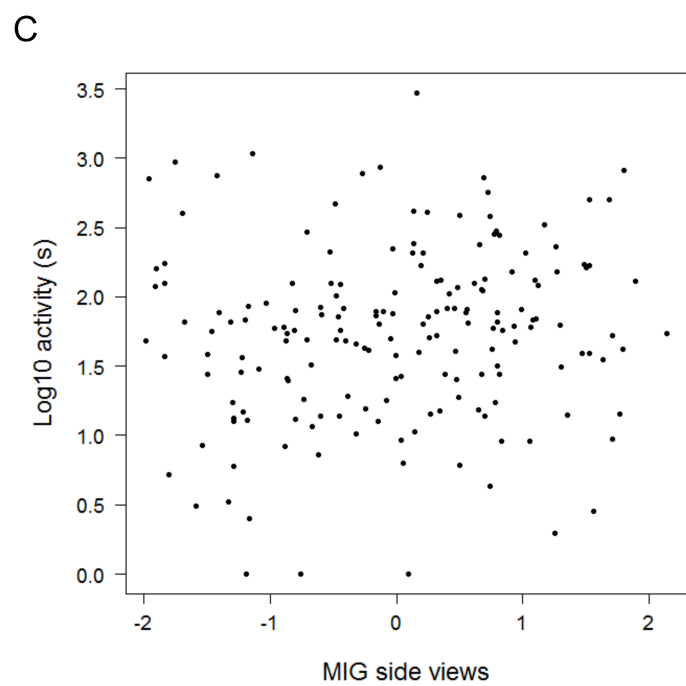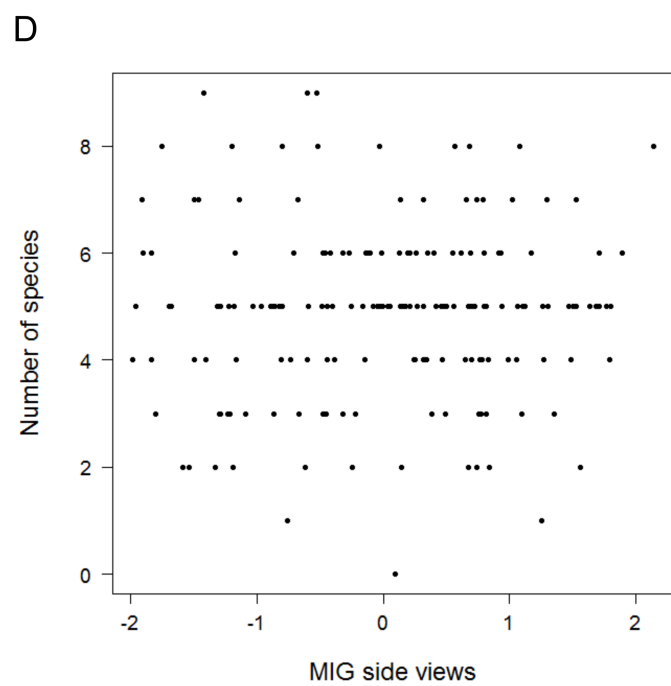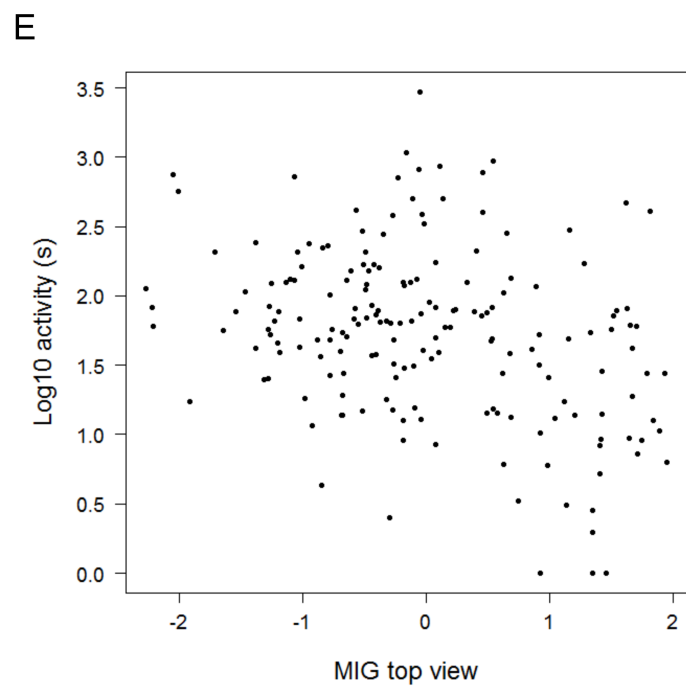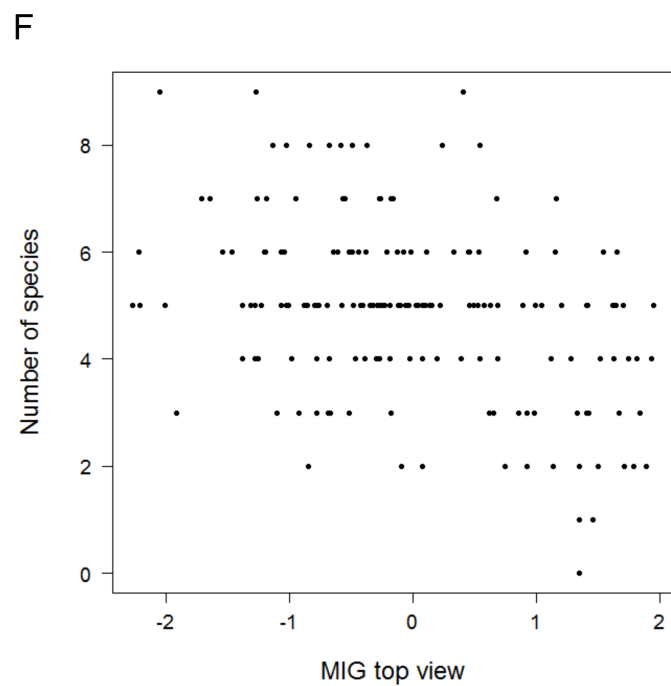

Supplement: Supplementary file 2 [file ECE3-8-3240-s002.pdf]

A

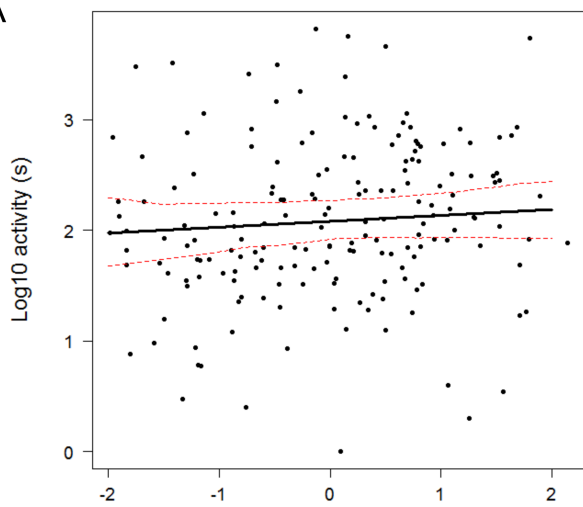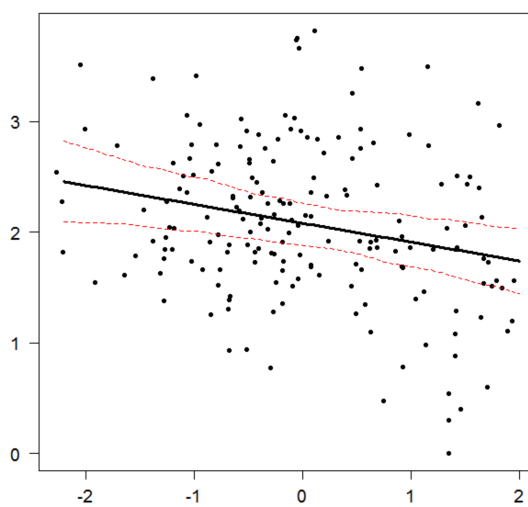

B

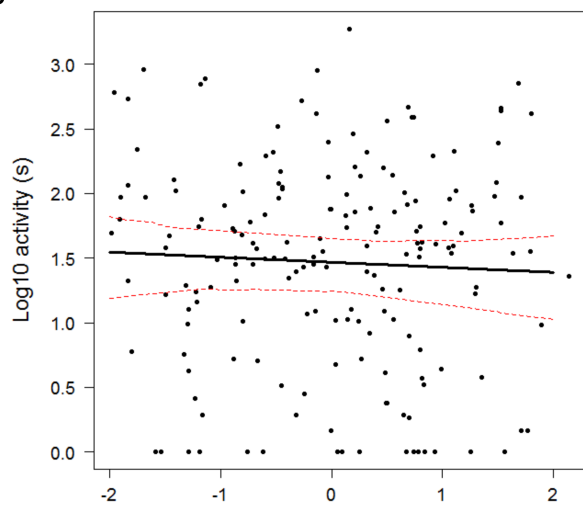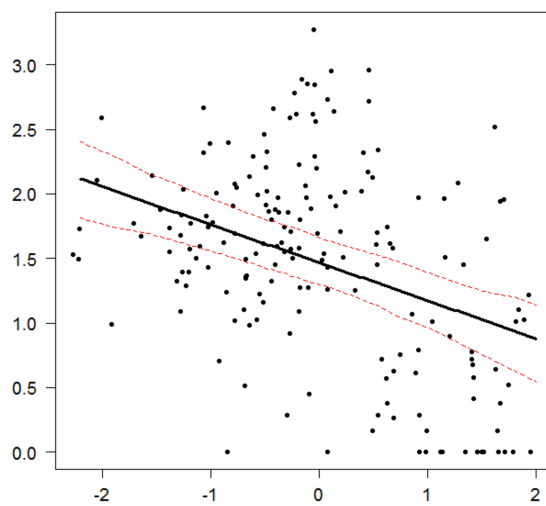

C

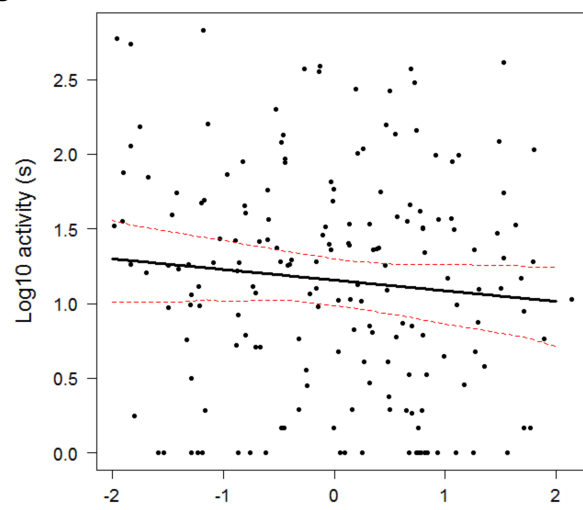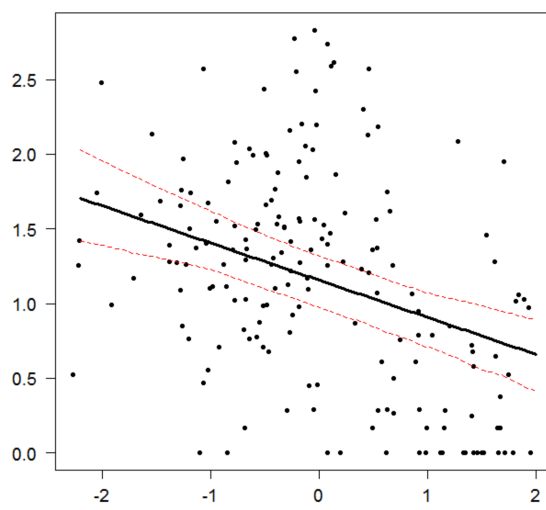

D

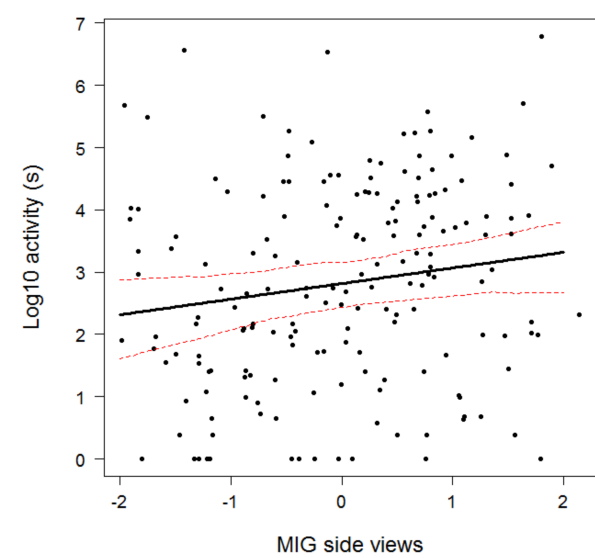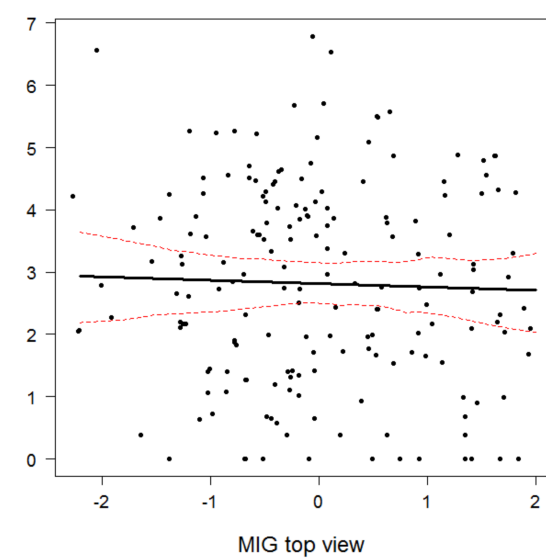

Supplement: Supplementary file 3 [file ECE3-8-3240-s003.pdf]
